# Gene activation regresses atherosclerosis, promotes health, and enhances longevity

**DOI:** 10.1186/1476-511X-9-67

**Published:** 2010-07-06

**Authors:** Pauli V Luoma

**Affiliations:** 1 Institute of Biomedicine, Pharmacology, University of Helsinki, Finland

## Abstract

**Background:**

Lifestyle factors and pharmacological compounds activate genetic mechanisms that influence the development of atherosclerotic and other diseases. This article reviews studies on natural and pharmacological gene activation that promotes health and enhances longevity.

**Results:**

Living habits including healthy diet and regular physical activity, and pharmacotherapy, upregulate genes encoding enzymes and apolipoprotein and ATP-binding cassette transporters, acting in metabolic processes that promote health and increase survival. Cytochrome P450-enzymes, physiological factors in maintaining cholesterol homeostasis, generate oxysterols for the elimination of surplus cholesterol. Hepatic CTP:phosphocholine cytidylyltransferase-α is an important regulator of plasma HDL-C level. Gene-activators produce plasma lipoprotein profile, high HDL-C, HDL_2_-C and HDL-C/cholesterol ratio, which is typical of low risk of atherosclerotic disease, and also of exceptional longevity together with reduced prevalence of cardiovascular, metabolic and other diseases. High HDL contributes to protection against inflammation, oxidation and thrombosis, and associates with good cognitive function in very old people. Avoiding unhealthy stress and managing it properly promotes health and increases life expectancy.

**Conclusions:**

Healthy living habits and gene-activating xenobiotics upregulate mechanisms that produce lipoprotein pattern typical of very old people and enhance longevity. Lipoprotein metabolism and large HDL_2 _associate with the process of living a very long life. Major future goals for health promotion are the improving of commitment to both wise lifestyle choices and drug therapy, and further the developing of new and more effective and well tolerated drugs and treatments.

## Introduction

It is common knowledge that diet, exercise and less stress are likely to enhance longevity, but the mechanisms are not well known. Chronic stress can lead to behavioral and somatic disorders such as anxiety, depression, obesity, dyslipidemia, metabolic syndrome, and atherosclerosis with its cardiovascular sequalae, premature cardiovascular disease and death [1]. On the other hand, prudent diet and regular physical activity, and pharmacological compounds, have metabolic effects that reduce the risk of cardiovascular and other diseases. Atherosclerotic cardiovascular disease is the leading global health problem as assessed by mortality [2], and the research focusing on the atherosclerotic vascular process is of particular importance for health promotion.

Population studies performed in the 1970s identified high plasma apolipoprotein AI (apo AI) and HDL cholesterol (HDL-C) levels as powerful indicators of a low risk of coronary heart disease (CHD) and cerebrovascular disease [3,4], whereas high apo B and LDL-C indicate an increased risk of CHD and death. The close relation of the major risk factors to cardiovascular diseases led to great continuing interest in agents with potential to raise apo AI and HDL-C and reduce apo B and LDL-C. Clinical studies soon revealed that drugs which induce protein synthesis increase hepatic cytochrome P450 activity and plasma apo A1 and HDL-C and the HDL-C/cholesterol ratio [5,6]. The inducing agents are gene-activators that can also upregulate hepatic LDL receptor (LDLR) gene and reduce plasma LDL-C [6,7]. These compounds influence cholesterol homeostasis also in other tissues including vasculature, intestine and the brain. A number of physiological and pharmacological compounds act, via the activation of enzyme, receptor and transporter genes, against the atherosclerotic process, whereas mutations in the genes can promote atherogenesis. The ability to control the expression of these genes provides a possibility to influence the development of cardiovascular disease and to extend lifespan. Investigations performed in the last few years focusing on this possibility have resulted in significant positive results. This article reviews studies on natural and pharmacological gene activation that regresses atherosclerosis, reduces the incidence of cardiovascular and other diseases, and enhances longevity.

## Liver and plasma lipids and proteins and gene-activation

**Figure 1:**
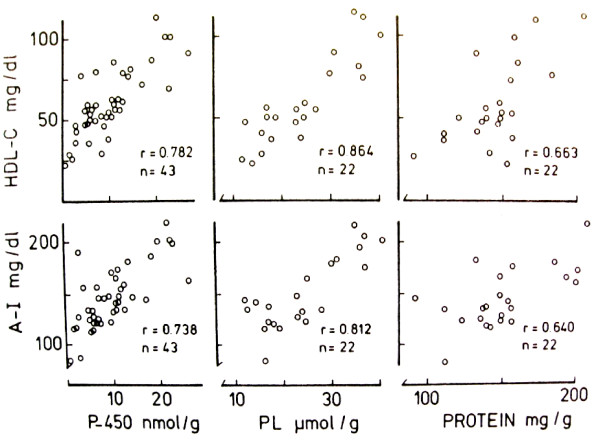
**Relation of plasma HDL-C and apolipoprotein AI to liver cytochrome P450, phospholipid (PL) and protein in man**. Some patients were treated with gene-activating drugs (Luoma PV el al, Lancet. 1982, **1**:625).

Both endogenous and exogenous factors act on the risk and the development of atherosclerosis. An endogenous induction of apo AI synthesis resulting in high plasma apo AI and HDL-C concentration has been identified as a cause of familial hyperalphalipoproteinemia which is characterized by a low death rate from CHD and prolonged life expectancy [8], while a low apo AI synthesis rate leads to hypoalphalipoproteinemia [9] that promotes atherogenesis. Similarly, drugs which induce hepatic protein and phospholipid synthesis and P450 activity, could increase plasma apo AI and HDL-C [10] (Figure 1) and the HDL-C/cholesterol ratio [11] that reflects an enhanced hepatic secretion of apo AI-phospholipid complexes. Corresponding to these effects, an overexpression of human apo AI transgene prevents atherogenesis [12] and an infusion of apo AI-phospholipid complexes in humans regresses atherosclerosis [13]. The liver produces the majority of HDL phospholipids, phosphatidylcholine (PC) being the primary phospholipid in cellular membranes and plasma lipoproteins [14]. Hepatic CTP: phosphocholine cytidylyltransferase-α (CCTα) is the key enzyme in the synthesis of PC and an important regulator of plasma HDL-C level (next chapter).

## Enzymes, atheroprotection and atherosclerosis

A number of enzymes participate in processes that protect arteries from atherosclerosis, including P450-enzymes belonging to different cytochrome (CYP) classes, CCTα, sirtuin 1(SIRT1) and paraoxonase-1 (PON1).

P450-enzymes have in addition to recognized pharmacological and toxicological functions, important physiological functions in the control of cellular homeostasis. P450-isoenzymes such as CYP7A1, CYP27A1 and CYP46A1 are natural regulators of cholesterol homeostasis which hydroxylate it to oxysterols (OHCs) [15,16]. CYP7A1 is essential for the conversion of cholesterol to 7α-OHC and as the key rate-limiting enzyme in the synthesis of bile acids. A mutation in CYP7A1gene can reduce bile acid synthesis, increase hepatic cholesterol, and present as a phenotype reminiscent of that in familial hypercholesterolemia (FHC) associated with premature atherosclerotic disease [17]. Correspondingly, a low rate of bile acid synthesis in FHC predicts enhanced mortality from CHD [18], and bile acid binding and CYP7A1 inducing resin therapy reduces cardiovascular mortality [19]. Side-chain OHCs such as 27OHC, 24SOHC and 25OHC generated by CYP27A1, CYP46A1 and cholesterol 25-hydroxylase, respectively, are signal mediators that have been identified as activators of liver X receptors (LXRs) both in vitro and vivo [20-22]. The OHCs influence cellular cholesterol homeostasis by serving as ligands for LXRs that upregulate genes acting in cholesterol elimination pathways, and through the suppression of SREBP (sterol regulatory element binding protein) maturation and promoting the degradation of hydroxymethylglutaryl CoA reductase (HMGCOAR), the rate-limiting enzyme of cholesterol synthesis [23]. CYP46A1 influences cholesterol homeostasis in the brain by converting it to 24SOHC, which readily passes the blood brain barrier and may also as a ligand for LXR promote the efflux of cholesterol. CYP3A4, a key enzyme in the metabolism of xenobiotics also affects the fate of cholesterol. CYP51A1 is the only P450 enzyme participating in cholesterol synthesis. The synthesis generates metabolites including, 24S,25 epoxycholesterol which suppresses SREBP-2 process and HMGCOAR activity, and may as a ligand for LXR prevent cholesterol accumulation [24].

Cardiovascular P450s include enzymes belonging to several CYP families [25-27]. Many of them generate eicosanoids that have important functions in vascular physiology. The CYP2C and 2J epoxygenases metabolize arachidonic acid to eicosanoids, epoxyeicosatrienoic acids (EETs) which are mediators in vascular signaling processes. CYP4 ω-hydroxylases convert EETs to dihydroxyeicosatrienoic acids (DHETs). Both EETs and DHETs activate peroxisome proliferator-activated receptors (PPARs), which have a role in vascular functions [26]. EETs are vasodilating compounds which inhibit inflammatory response, promote fibrinolysis and may function as endogenous antiatherogenic compounds [25,27]. A mutation in CYP2J2 gene has been linked to reduced plasma EET concentrations and increased risk of CHD [27]. CYP4A and CYP4F enzymes convert arachidonic acid to 20-hydroxy - eicosatetraenoic acid (20HETE) [26]. Vascular ω-hydroxylases further metabolize 20HETE to 20-carboxy-arachidonic acids which also activate PPARα and PPARγ receptors [27]. Several drugs including statins and fibrates are PPAR activators [25,28].

CCTα, the rate-limiting enzyme in the synthesis of PC, has been identified as a key player in maintaining plasma HDL-C levels [14]. CCTα knock-out mice show reduced hepatic PC content as well as ABCA1 (ATP-binding cassette A1) expression in hepatocytes and marked decrease in HDL-C [14]. Cholesterol and PC efflux to apo AI is impaired by CCTα deficiency in the hepatocytes [14]. Adenoviral delivery of CCTα to knock-out mice restored PC mass in knock-out livers, increased hepatic ABCA1 levels, and normalized HDL-C levels in vivo [14]. CCTα is also necessary for protection against cholesterol-induced cell death [14]. An elevation of cellular cholesterol induces the generation of side-chain OHCs that upregulate CCTα and PC synthesis [29]. Relatively low concentrations of OHCs increase CCTα activity and PC synthesis in adose-dependent manner.The OHCs could directly act on CCTα and increase PC synthesis, and thereby act against cholesterol toxicity [29]. The study of Gehrig et al. showed that direct activation of CCTα by OHCs is a potential mechanism to maintain sterol/phospholipid content of membranes within normal boundaries [29].

SIRT1 is a nicotinamide adenine dinucleotide-dependent deacetylase that regulates physiological processes including systemic and hepatic glucose, lipid and cholesterol homeostasis [30,31]. SIRT1 has broad biological functions in growth regulation, stress response, tumorigenesis, endocrine signaling, and extended lifespan [32]. The enzyme is connected to important metabolic changes and regulatory proteins in response to caloric restriction. Fasting increases SIRT1 activity that reduces insulin secretion and increases insulin sensitivity, stress resistance and cell survival [32]. Hepatic SIRT1 controls the expression of genes involved in cholesterol metabolic pathways [30]. It upregulates, via LXR activation, ABCA1 transporter and cellular cholesterol efflux to apo AI and HDL formation [31]. The expression of ABCA1 is reduced in SIRT1 knockout mice, resulting in defective cholesterol efflux and lower HDL-C levels, and blunted response to LXR agonists [31]. SIRT1, which extends life span in several species including mammals has emerged as a drug development target for treating age-dependent diseases [31-33].

PON1, an enzyme with antioxidant function primarily synthesized in the liver, is associated with HDL in the subintimal space of the artery. HDL together with PON1 is ideally positioned to protect LDL from oxidative modification in the arterial wall and to prevent the processes that initiate atherogenesis. Lifestyle factors, a number of diseases, drugs and nonpharmacologic factors may influence PONI 1 activity (reviewed in ref. 34). An atherogenic diet and smoking decrease PON1 activity, and it is low in patients with surgical menopause, type 1 diabetics with elevated blood glucose, chronic hepatitis and cirrhosis, rheumatoid arthritis and Alzheimer's disease. On the other hand, exercise, moderate alcohol consumption, hormone replacement therapy, and the use of statins, fibrates or aspirin associates with increased PON1 activity [34]. Patients with a mutated PON1 gene show accelerated atherosclerosis as assessed by carotid intima-media thickness (IMT) [35], and two prospective studies associated low PON1 activity with high incidence of major cardiovascular events [34]. An overexpression of human PON1 in mouse models of atherosclerosis decreased aortic lesion size [34].

## Receptors, transcription factors, and risk factors

Nuclear receptors regulate the expression of genes encoding enzymes, transporters and other proteins involved in metabolic homeostasis [36]. LXR receptors (LXRα and LXRβ) function as cholesterol sensors and mediators that act on genes involved in the absorption, efflux, and transport of cholesterol to the liver, bile acid synthesis, biliary sterol secretion, and the synthesis of nascent HDL [37]. LXR activation downregulates intestinal Niemann-Pick C1 like transporter which is critical for cholesterol absorption [38], and has similar effect on CYP51A1 and squalene synthase, key enzymes in the synthesis of cholesterol [39]. PPAR receptors, PPARα, PPARγ and PPARδ, control lipid and glucose metabolism, as well as depress the inflammatory response in various tissues and cells including macrophages and monocytes [36,40]. PXR is a regulator of xenobiotic metabolizing P450 enzymes which hydroxylate cholesterol and its metabolites [41]. PXR also is a positive regulator of CYP27A1 in human intestinal cells and could via LXRα activation stimulate cholesterol efflux from intestinal cells to apo AI and HDL formation [42].

SREBPs are transcription factors that activategenes dedicated to the synthesis and uptake of cholesterol, fatty acids, phospholipids and triglycerides [7]. SREBP-2 preferentially activates genes involved in the synthesis of cholesterol. LDLR is a membrane cholesterol transporter that mediates cellular uptake of LDL-C. A depletion of hepatic cholesterol activates SREBP processing that induces the expression of LDLR. Scavenger receptor B1 (SR-B1) is a cell-surface HDL receptor expressed in several tissues including the liver [43]. It facilitates cholesterol efflux from peripheral cells to HDL and mediates the hepatic uptake and biliary secretion of cholesterol. Studies using SR-B1- transgenic and - deficient mouse models have established the antiatherogenic activity of SR-B1 in vivo.

## ABC-transporters, apolipoproteins, HDL and cholesterol

ABC transporters have a major role in the control of whole-body cholesterol homeostasis [44]. ABCA1 regulates the rate-limiting step in the biogenesis of HDL particles by mediating the efflux of cholesterol and phospholipids across cellular membranes [44-46]. Hepatic ABCA1 is the main factor in the generation of HDL-C levels in the circulation. ABCG1 which is expressed in several tissues facilitates cholesterol efflux to HDL. ABCG4 transporter acts on cholesterol in the brain, and ABCG5 and ABCG8 in the liver and intestine. ABCA7 may mediate HDL assembly and generate cholesterol-poor HDL [47]. Apo AI, the predominant protein constituent of HDL, is produced by liver and intestine. It removes cellular cholesterol and phospholipids transported to plasma membrane [44]. Lipid-free and lipid-poor apo AI are the key molecules in the initiation of HDL formation. Apo AI is an activator of lecithin: cholesterol acyltransferse (LCAT) and a ligand for the binding of HDL to SRB1. Other apolipoproteins including AII, E, CI, CII, CIII, and AIV also serve in ABCA1-mediated cholesterol efflux [44].

## Genes, familiality, risk factors and lifespan

The contribution of genetic factors to life expectancy has become increasingly important at advanced ages [48]. Exceptional longevity is also familial. People in the most advanced decades of life, especially centenarians, possess lipoprotein profile that is typical of low risk of atherosclerotic disease. They have high HDL-C and HDL_2_-C [49], and also high HDL-C/cholesterol ratio [49,50] and low cholesterol and LDL-C as compared to septuagenarians [50,51]. Large HDL, i.e. the subclass HDL_2_, and large LDL have been identified as typical lipoproteins of exceptional longevity [52]. The predominance of large lipid-rich HDL_2 _subclass is the most reproducible phenotype among very old people [49], who also have reduced prevalence of cardiovascular diseases, hypertension, metabolic syndrome and diabetes mellitus [48,49,52]. The offsprings of very old people also have lower prevalence of cardiovascular disease and cardiovascular risk factors and a greater survival rate when compared to the controls who had one parent die at average life expectancy [53,54].

## Diet - gene activation, risk factors, and health effects

Dietary factors have significant metabolic and health effects. Diet constituents such as soy proteins and alcohol induce the expression of apo AI gene and raise HDL-C, and vitamins, e.g. vitamin C and E might have similar effect [55]. Vitamin B_3 _or niacin has recently been shown to increase apo AI production in man [56] and vitamin A deritative 9-*cis *retinoid acid to upregulate apo AI gene expression in HepG2 cells [55,57]. Weight control by a low calorie diet with adequate nutrition has positive effects. It increases insulin sensitivity, stress resistance, and cardioprotection, and decreases insulin secretion, fat storage and glycolysis [32]. The activating effect of the diet on SIRT1 could contribute to atheroprotection. The enzyme is positive regulator of LXR and cholesterol efflux via HDL formation. SIRT1 overexpression in fasted state could also induce hepatic CYP7A1 and decrease hepatic cholesterol content [30,33] that upegulates LDLR pathway and reduces plasma LDL-C.

Natural mechanisms protect cells from harmful effects of the diet. An atherogenic cholesterol-rich diet upregulates hepatic P450-enzymes that reduce cholesterol toxicity by inducing its catabolism to OHCs and elimination [58], and an activation of hepatic LXRα to protects animals from atherosclerosis of dietary origin [59]. Fatty acids and eicosanoids are endogenous activators of PPARs that also respond to high fat diet and act against atherogenic dyslipidemia and promote reverse cholesterol transport [36]. PXR mediates the activation of hepatic cholesterol metabolizing P450-enzymes and thereby protects against acute toxicity caused by a high-cholesterol diet [41].

Randomized trials show that modest consumption of oily fish, 1 to 2 servings per week, reduces CHD mortality by more than one third, with 17% reduction in total mortality, and indicate consistent and substantial reductions in cardiovascular risk related to lower trans fat consumption, consumption of whole grains, legumes, and cereal fiber, and consumption of fruits and vegetables [60]. Great improvements can be reached by promoting Mediterranian-type of diet [61] which is characterized by high intake of olive oil, vegetables, legumes, fruits nuts and cereals, a moderately high intake of fish, but low intake of saturated lipids, meat and poultry, and a regular but moderate intake of ethanol, primarily in the form of wine and generally during meals [62]. The people on Mediterranian diet have a favorable cardiovascular risk factor profile, low systolic and diastolic blood pressure, and fasting blood glucose and insulin levels [63], together with low mortality rate from CHD, cardiovascular disease, cancer, and also all-cause mortality [62-64].

## Physical activity - metabolic and health effects

Regular physical activity has multiple metabolic and health effects (Table 1) [60]. It reduces plasma LDL-C, cholesterol and triglycerides, and raises HDL-C and HDL_2_-C [65]. It also upregulates LXRα, PPARγ, and scavenger receptor CD36 (cluster of differentiation 36) that leads to an increased uptake of atherogenic oxidized LDL, and ABCA1 and ABCG1 in leucocytes [66]. Endurance-trained men show high VO_2_max (peak pulmonary oxygen uptake) together with high HDL-C, apo AI, and increased plasma LCAT activity and cholesterol efflux from macrophages compared with normally active men [67,68]. The increased LCAT activity leads to an increased production of HDL_2 _[69]. Experimental studies also show that endurance exercise upregulates hepatic P450, LDLR and SR-B1, increases the catabolism of cholesterol to bile acids, and prevents gallstone formation [70]. In addition, regular physical activity helps maintaining weight control, improves glucose - insulin homeostasis, lowers blood pressure, and improves psychological wellbeing [60]. It also reduces, in proportion to overall physical activity, the mortality form CHD, cardiovascular disease, and cancer, and also from any cause [71,72]. Regular physical exercise also facilitates smoking cessation that reduces total mortality by approximately one third [60].

**Table 1:** Effects of regular aerobic physical activity^1^

**Upregulation**	
**Receptors**	LXRα, PPARγ SR-B1, LDLR, CD36
**Enzymes**	CYPs, LCAT, PON1
**Transporters**	ABCA1, ABCG1, Apo AI
**Change in risk factor**	
**Increase**	Apo AI, HDL-C, HDL_2_-C
**Decrease**	LDL-C, cholesterol, triglycerides
**Improvement**	cellular cholesterol efflux
	peak pulmonary oxygen uptake
	glucose - insulin homeostasis
	weight control
	psychological wellbeing
**Lowering**	blood pressure
**Decrease**	CHD, cardiovascular, cancer and total mortality

^1 ^The list of abbreviations precedes the reference list.

## Stress - mediators and diseases

The main central molecular mediators of the stress system are corticotropin-releasing hormone, arginine vasopressin and norepinephrine, and key peripheral mediators are corticotropin, cortisol, arginine vasopressin, norepinephrine, epinephrine and interleukin-6 [1]. The stress system is activated in a coordinated fashion during acute stress influencing central and peripheral functions that are important for adaptation and survival. Chronic activation of stress system is associated with many negative manifestations. It can lead to common mental and somatic disorders such as anxiety, depression, and development of obesity and metabolic syndrome with insulin resistance, dyslipidemia, arterial hypertension, type 2 diabetes, and atherosclerosis ultimately resulting in premature cardiovascular disease and death [1].

## Studies on model organisms and longevity

Experiments in model organisms such as Caenorhabditis (C) elegans and Drosophila (D) melanogaster have identified a number of potential genetic mechanisms which can increase survival. The genes involved include daf-2, an insulin receptor-like gene, and daf-12, a nuclear receptor gene in C. elegans, and catalase and suproxide dismutase genes in D. melanogaster. Experiments in these organisms have demonstrated that changes in genes can markedly increase lifespan, but to what extent they explain variation in humans is still uncertain [73]. The environments in which the genes come to expression in these species and humans are markedly different, and caution should be taken in extrapolating the relevance of results on genetic variation in model organisms to the human situation [73].

## Xenobiotic gene-activators - antiatherogenic effects

A number of xenobiotics are gene-activators that have antiatherosclerotic actions. Several of them, but not all, are P450 inducers.The major effects of statins, niacin, fibrates and cholestyramine, important drugs for treatment of dyslipidemias, are presented here. Recent studies have contributed significant positive results. Other compounds with beneficial effects include angiotensin-converting enzyme inhibitors (ACEIs), calcium channel blockers (CCBs), angiotensin receptor blockers (ARBs), glitazones, anticonvulsants, retinoids and alcohol [74].

Statins inhibit HMGCOAR and cholesterol synthesis and decrease hepatic cholesterol that activates SREBP processing and upregulation of LDLRs on the surface of liver cells and reduces plasma LDL-C [7]. The drugs also induce PPARα and apo AI synthesis in liver cells [58] and raise plasma apo AI, HDL-C and the subfraction HDL_2_-C [75]. Statins also stimulate CYP4 ω-hydroxylases in human liver microsomes and CYP2 epoxygenase in native endothelial cells which generate PPAR-activating eicosanoids [25,28]. The drugs upregulate via PPAR activation LXR, ABCA1 and ABCG1 genes in cholesterol-loaded macrophages and hepatocytes, and promote cholesterol efflux to apo AI and HDL [58]. Statins also induce, through SREBP-2 activation, CYP4F2 [76] and ABCA1 [77] genes in human hepatocytes.

Niacin, which is not a P450-inducer, has recently been shown to stimulate apo AI production in patients with combined hyperlipidemia [56]. The drug also upregulates PPARγ, LXRα and ABCA1 genes and promotes HDL-dependent cholesterol efflux from monocytoid cells [78], and raises apo AI and HDL-C, particularly HDL_2_-C [79], and reduces LDL-C and triglycerides. Statin - niacin combination is more effective than monotherapy in raising HDL-C and HDL_2_-C and reducing LDL-C and triglycerides [80]. Fibrates, PPARα agonists which induce P450s including ω-hydroxylases and apo AI synthesis [58], raise plasma apo AI and HDL-C and reduce LDL-C and triglycerides. Fibrate-caused PPARα activation upregulates LXR, SR-B1 and ABCA1 genes and promotes apo AI-mediated cholesterol efflux [40]. Cholestyramine binds bile acids, induces CYP7A1 and depletes cholesterol in hepatocytes, and increases LDL-C elimination via the upregulated LDLR pathway, and also induces apo AI synthesis and raises plasma apo AI and HDL_2_-C subfraction [81]. The combination of resin and statin is effective in reducing circulating cholesterol levels [82].

## Xenobiotic gene-activators, diseases and longevity

Several xenobiotic gene-activators prevent or regress atherosclerosis and increase survival. Recent studies have contributed significant positive results. The major effects of statins, niacin, fibrates, cholestyramine, ARBs, ACEIs and CCBs are presented here. Other compounds including pioglitazone, anticonvulsants and alcohol also have positive effects [74].

Statins including pitavastatin [83], fluvastatin [84], pravastatin [85], rosuvastatin, atorvastatin and simvastatin [58], regress coronary atherosclerosis as assessed by intravascular ultrasonography (IVUS) technique. Statin therapy that caused a moderate rise in HDL-C level together with significant LDL-C lowering was particularly effective in regressing atherosclerotic plaque [86]. The statins also reduce both nonfatal and fatal cardiovascular events, and all-cause mortality [58]. The drugs improved survival rate also of people with cardiovascular risk factors but without cardiovascular disease [87], and population studies linked statin therapy with reduced mortality rate chronic obstructive pulmonary disease and pneumonia [88]. A comparison of mortality of statin users (n = 228,528) versus nonstatin users (n = 1,261,938) in the United States veteran population revealed a highly significant increase in survival rate [89]. Almost half of statin users had started the therapy at the age of 70 years or older, and the conclusion was that the therapy is an effective strategy for promoting longevity.

Niacin added to statin therapy regressed atherosclerosis in carotid arteries as measured by IVUS [90], and a follow-up trial revealed increased survival rate for individuals initially randomized to niacin [58]. Fibrates prevent the progression of coronary atherosclerosis as assessed by angiography [58]. A ten-year fibrate therapy for dyslipidemia increased survival rate as compared with dyslipidemic persons without treatment [91], and a follow-up control of the Helsinki Heart study revealed that gemfibrozil reduces CHD mortality, and also total mortality of obese persons with high triglycerides [92]. Cholestyramine therapy had positive effect on coronary atherosclerosis assessed by angiography and it reduced the risk of CHD death and/or nonfatal myocardial infarction [93], and resin therapy, cholestyramine or colestipol, reduced cardiovascular mortality [19].

ACEIs, ARBs and CCBs, drugs indicated for cardiovascular diseases, also have antiatherosclerotic effects. ACEI treatment has been shown to reduce carotid IMT [94] and ARB treatment plaque volume in coronary arteries [95], and CCB therapy to widen the lumen and inhibit the intimal hyperplasia in coronary stent lesions [96]. The antiatherosclerotic effects could contribute to the improved survival rate reported for ACEI- [97] and CCB- [98] treated subjects, and to reduced rate of cardiovascular events of ARB-treated individuals [97].

## Goals for health promotion

The effects of healthy lifestyle and pharmacotherapy, the decrease in the incidence of cardiovascular and other diseases and the increase in life expectancy, emphasize the importance of future actions that improve commitment to both healthy living habits and drug therapy, and further on developing of new and more effective and well tolerated drugs and therapies. Poor dietary habits, unhealthy composition of diet with excess calories, inadequate physical activity and adiposity, smoking, and excess stress, are major problems that affect health in modern societies. Hence, the clinical evaluation and treatment of dietary, physical activity, and other living habits must become as routine and familiar as assessment of blood lipid and glucose levels, and blood pressure. It is also important to avoid unhealthy stress and to learn to how to manage it. Effective focusing on life style risk prevents or delays the occurrence of CHD, cardiovascular disease, diabetes mellitus and obesity, and reduces the need of drug therapy or even makes it unnecessary.

The positive effects of gene activators have led to intensive search for new molecules with potential effects on risk factors and effectors in the antiatherogenic metabolic cascade, such as enzymes, receptors and transporters. HDL and its constituents are key therapeutic targets for preventing atherosclerotic lesion formation and promoting the regression of established atherosclerotic lesions [99]. Many P450-inducing compounds increase apo AI, HDL-C and the HDL-C/cholesterol ratio [5,6], and new molecules acting on cholesterol metabolizing P450-enzymes in the liver, brain and other tissues are also potentially effective antiatherogenic agents. Investigational agents also include new LXR agonists and PPAR agonists and other HDL elevators, and compounds enhancing the expression of ABC transporters [74]. The agonists of PXR, which affects the fate of cholesterol, also have potential to activate antiatherogenic mechanisms. The evaluation of the effects of the combinations lifestyle factors and drug treatments and of drug combinations can significantly improve therapeutic possibilities.

## Discussion

The reviewed studies show that living habits and pharmacological compounds upregulate genetic mechanisms that beneficially affect lipoprotein metabolism, promote wellbeing and increase survival. The studies also associate lipoprotein metabolism and HDL_2 _subclass with the process of living a very long life. The liver is the principal site for the synthesis of lipids and proteins, and changes in hepatic function can significantly affect lipoprotein metabolism and the development of atherosclerotic and other diseases.

The first studies clarifying the effects of genes on hepatic proteins and P450 activity and plasma apolipoprotein and lipoprotein risk factors were performed in the 1970s. Our original observations linked gene activation and P450 induction with increase in hepatic protein and phospholipid concentration and with cardiovascular risk factor profile that is typical of low risk of atherosclerotic disease [5,6], and that later on has been found typical also for very old people together with decreased incidence of cardiovascular, metabolic and other diseases. A number of gene-activators including statins, niacin, cholestyramine, nifedipine [100], pioglitazone [101] and phenytoin [102], and also alcohol consumption [103], increase HDL-C and the large, PC-rich HDL_2_-C, and particularly statins, niacin and cholestyramine are effective in reducing cholesterol and LDL-C. HDL_2 _-C also rises with ageing and is typical for very old people [49,52].

**Figure 2:**
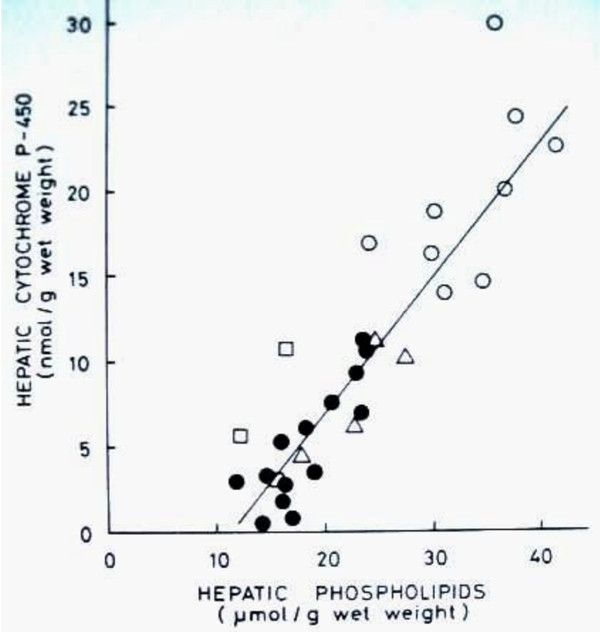
**Relationship between cytochrome P450 and phospholipid concentrations in human liver**. Δ patients with normal liver histology, ○ patients with normal liver but undergoing inducing drug therapy, ● patients with fatty liver, □ patients with liver cirrhosis. r = 0.909, p < 0.001 for all subjects. For fatty liver group, r = 0.834, p < 0.001 (Savolainen MJ et al, Eur J Clin Pharmacol. 1985, **27**:727-732).

Several gene-inducing mechanisms are active in the prevention and regression of atherosclerosis. Both lifestyle factors and pharmacological compounds upregulate apo AI and LDLR gene expression and raise HDL-C and and reduce LDL-C, respectively. They similarly activate several genes in the cholesterol elimination pathways, including nuclear receptors, ABC transporters, and enzymes such as CYPs and CCTα. Many gene-activators increase the subfraction HDL_2 _which has high apo AI and phospholipid content compared with HDL_3_, and key role in the antiatherogenic effect of HDL [104]. The individuals with high P450-activity in the liver, show high plasma HDL-C, HDL_2_-C and HDL-C/cholesterol ratio, while a deficient P450-activity leads to cholesterol accumulation, hypercholesterolemia and xanthoma formation and promotes atherogenesis [11,105]. The increase of HDL-C and HDL_2_-C with increasing hepatic P450 activity suggests that the cholesterol metabolizing CYPs, that are particularly prevalent in the liver [44], enhance the generation of OHCs and activation of HDL and HDL_2 _raising mechanisms. The relation of plasma HDL-C (Figure 1) [10,11] and liver P450 (Figure 2) [106] to hepatic phospholipids also suggests that the enrichment of PC in HDL_2 _with increasing P450 activity reflects the effect of these CYPS on the synthesis of PC, the primary phospholipid of HDL. An effective CYP-mediated OHC generation could induce hepatic CCTα and PC synthesis [29], and consequently increase HDL-C, and particularly the PC-rich HDL_2_-C. A similar effect of a P450-inducing agent on OHC production and hepatic PC synthesis could also have a role in the elevation of HDL-C and HDL_2_-C. The studies on CCTα-deficient mice showing a decrease of PC content in the liver, the principal source of PC in HDL, as well as ABCA1 expression in hepatocytes and marked lowering of HDL-C levels [14], support this possibility. Correspondingly to these effects, a delivery of CCTα to knock-out mice increased hepatic PC mass and ABCA1 levels, and plasma HDL-C levels in vivo. In agreement with these findings, the high phospholipid content in HDL_2 _particles has been identified as an efficient driving factor for cholesterol removal from peripheral cells [107,108], and HDL phospholipids correlate inversely with the severity of angiographically defined coronary atherosclerosis [109].

The OHCs mediate the activation of antiatherogenic mechanisms. They upregulate, via LXR, effectors in cholesterol efflux including ABCA1, ABCG1 and apo AI. An elevation of cellular cholesterol upregulates hepatic P450-enzymes, OHCs [110], CCTα [29], and ABCA1, and increases plasma HDL-C [111], whereas targeted deletion of hepatic ABCA1 markedly reduces it [112]. Corresponding to this effect, a recent study of Brunham et al. showed that mice lacking hepatic ABCA1 display a significant accumulation of cholesterol in aorta and increase in aortic atherosclerotic lesion area, while a selective deletion of macrophage ABCA1 did not significantly modulate atherogenesis [113]. These studies on hepatic enzymes, mediators, lipids and transporters, and atherosclerosis, emphasize the key role of liver in maintaining cholesterol homeostasis and in the prevention and regression of atherosclerosis.

Gene-activators increasing HDL and apo AI have, in addition to cholesterol efflux and transport, a number of other favorable effects (Table 2) [6,114]. They inhibit inflammatory processes in vascular cells, promote endothelial cell nitric oxide (NO) release and vasodilatation, and have a variety of antioxidative actions. HDL together with PON1 and other enzymes inhibit the atherogenic oxidative modification of LDL in the arterial wall. Other antiatherogenic actions of HDL/apo AI include inhibition of platelet aggregation, stimulation of prostacyclin production and fibrinolysis, and inhibition of expression adhesion molecules and apoptosis of endothelial cells. Interestingly, high plasma HDL and apo AI also associate with good cognitive performance in very old people [49,115], suggesting that the antiaggregation, anti-inflammatory and antioxidative effects HDL/apo AI have a role in this relation.

**Table 2:** Antiathrogenic effects of HDL/apolipoprotein AI

**Efflux and reverse transport of cholesterol**	
**Anti-inflammatory**	
	- inhibition of the expression of vascular cell adhesion molecules
	- stimulation of endothelial NO production
**Antioxidative**	
	- inhibition of oxidative modification of LDL
**Antithrombotic**	
	- inhibition of platelet aggregation
	- fibrinolysis
	- increase of prostacyclin production
**Facilitation of endothelial cell function and repair**	
**Inhibition of apoptosis of endothelial cells**	

## Conclusions

The studies performed in the 1970s and thereafter have demonstrated that living habits and pharmacological compounds upregulate genetic mechanisms which act in maintaining cellular homeostasis, have beneficial metabolic and health effects, and enhance longevity. Major findings of the investigations associate gene activation and hepatic protein and phospholipid metabolism with plasma lipoprotein profile, high HDL-C, HDL_2_-C and HDL-C/cholesterol ratio, that is typical of low risk of cardiovascular and other diseases, and also of exceptional longevity. In addition, high HDL contributes to protection against oxidation, inflammation and thrombosis, and associates with good cognitive function in people of advanced age.

The studies on nuclear receptors, enzymes, phospholipids, proteins and ABC transporters indicate that the liver has a major role in atheroprotection and regression of atherosclerosis. P450-enzymes, physiological key factors in maintaining cholesterol homeostasis generate oxysterols for the removal of excess cholesterol. Hepatic CCTα is the key enzyme in the synthesis of PC and an important regulator of plasma HDL-C level, and the increased phospholipid mass in HDL_2 _is an efficient driving factor for cholesterol removal from peripheral cells. Healthy lifestyle choices counteract, via natural gene activation, the atherosclerotic vascular process and increase survival, and gene-activating xenobiotics upregulate mechanisms that produce lipoprotein pattern typical of very old people together with reduced incidence of cardiovascular, metabolic and other diseases. Avoiding unhealthy stress and managing it properly promotes health and increases life expectancy.

The positive effects of living habits and pharmacological compounds on the incidence of cardiovascular, metabolic and other diseases, and life expectancy, emphasize the importance of future activities that increase the adherence to both healthy lifestyle and drug treatment, and further of searching of new, effective and well tolerated agents and therapies.

## Abbreviations

ABC: ATP-binding cassette; ARB: angiotensin receptor blocker; CCB: calcium channel blocker; CCTα: CTP:phosphocholine cytidylyltransferase-α; CD36: cluster of differentiation 36; DHET: dihydroxyeicosatrienoic acid; EET: epoxyeicosatrienoic acid; HETE: hydroxyeicosatetraenoic acid; HMGCOAR: hydroxymethylglutaryl-coenzyme A reductase; IMT: intima-media thickness; LCAT: lecithin:cholesterol acyltransferase; LXR: liver X receptor; PC: phosphatidylcholine; PPAR: peroxisome proliferator-activated receptor; PXR: pregnane X receptor; SR-B1: scavenger receptor B1; SREBP: sterol regulatory element binding protein

## Competing interests

The author declares that he has no competing interests.
